# Morphometric study of the primary ossification center of the frontal squama in the human fetus

**DOI:** 10.1007/s00276-020-02425-7

**Published:** 2020-02-05

**Authors:** Magdalena Grzonkowska, Mariusz Baumgart, Mateusz Badura, Marcin Wiśniewski, Michał Szpinda

**Affiliations:** Department of Normal Anatomy, The Ludwik Rydygier Collegium Medicum in Bydgoszcz, The Nicolaus Copernicus University in Toruń, Toruń, Poland

**Keywords:** Frontal bone, Bone development, Osteogenesis, Fetal development

## Abstract

**Purposes:**

Detailed morphometric data on the development of ossification centers in human fetuses is useful in the early detection of skeletal dysplasias associated with a delayed development of ossification centers and their mineralization. Quantitative analysis of primary ossification centers of cranial bones is sporadic due to limited availability of fetal material.

**Material and methods:**

The size of the primary ossification center of the frontal squama in 37 human (16 males and 21 females) spontaneously aborted human fetuses aged 18–30 weeks was studied by means of CT, digital-image analysis and statistics.

**Results:**

With neither sex nor laterality differences, the best-fit growth dynamics for the primary ossification center of the frontal squama was modelled by the following functions: *y* = 13.756 + 0.021 × (age)^2^ ± 0.024 for its vertical diameter, *y* = 0.956 + 0.956 × age ± 0.823 for its transverse diameter, *y* = 38.285 + 0.889 × (age)^2^ ± 0.034 for its projection surface area, and *y* = 90.020 + 1.375 × (age)^2^ ± 11.441 for its volume.

**Conclusions:**

Our findings for the primary ossification center of the frontal squama may be conducive in monitoring normal fetal growth and screening for inherited faults and anomalies of the skull in human fetuses.

## Introduction

In the prenatal period, an important element of routine ultrasound examinations is the assessment of the fetal facial skeleton, as facial dysmorphia is a common phenomenon in genetic syndromes and chromosomal defects. Moreover, using 3D-ultrasound, it is possible to reliably assess individual facial bones along with sutures [4].

Quantitative analysis of the ossification centers of skull bones are very rare due to limited availability of fetal material [16], but still detailed morphometric data on the development of ossification centers in human fetuses is useful in the early detection of skeletal dysplasias associated with a delayed development of ossification centers and their mineralization [21]. Numerous congenital defects, including Hajdu–Cheney syndrome, Down’s syndrome, cleidocranial dysplasia, pycnodysostosis and craniosynostosis involve the underdevelopment of the nasomaxillary region, which is often associated with reduced maxillary sinus pneumatization and failed closure of the frontal suture [17].

Knowledge of the growth of individual skull bones in human fetuses can be useful in such fields as anatomy, anthropology, orthodontics, radiology, obstetrics, pediatrics, orthopedics and facial reconstruction surgery [14].

Although the timing of ossification of each bone forming the skull is known, no morphometric measurements of the primary ossification center of the frontal squama have been reported. This is the first report in the literature regarding the morphometric analysis of the primary ossification center of the frontal squama in human fetuses based on computed tomography imaging.

In the present study we aimed:

- to determine normative age-specific values for linear, planar and volumetric parameters of the primary ossification center of the frontal squama in human fetuses;
- to examine possible sex differences for all analyzed parameters;
- to compute growth dynamics for the analyzed parameters, expressed by best-matched mathematical models.

## Material and methods

The study material comprised 37 human fetuses (16 males and 21 females) aged 18–30 weeks of gestation, originating from spontaneous miscarriages and preterm deliveries. The fetuses were collected before the year 2000 and still remain part of the fetal collection of the Department of Normal Anatomy. The experiment was approved by the Bioethics Committee of The Ludwik Rydygier Collegium Medicum in Bydgoszcz (KB 275/2011). The inclusion criteria of the fetuses studied were based on the evaluation of their explicit morphology and statistical cards with the course of pregnancy. Since on macroscopic examination neither internal nor external conspicuous morphological malformations were found, all included specimens were identified as normal. Of note, the fetuses did not display any developmental abnormalities of the musculoskeletal system. The fetal ages were determined on the crown-rump length and the known date of the beginning of the last maternal menstrual period. Furthermore, the fetuses studied could not suffer from growth retardation, as the correlation between the gestational age based on the crown-rump length (CRL) and that calculated by the last menstruation reached the value *R* = 0.98 (*p* < 0.001). Table 1 lists the characteristics of the study group, including age, number and sex of the fetuses.

**Table 1:** Age, number and sex of the fetuses studied

Gestational age	Crown-rump length (mm)				Number	Sex	
					of fetuses		
	Mean	SD	Min	Max	*N*	♂	♀
18	133.33	5.77	130.00	140.00	3	1	2
19	146.50	2.89	143.00	150.00	4	2	2
20	161.00	2.71	159.00	165.00	4	2	2
21	173.67	2.31	171.00	175.00	3	2	1
22	184.67	1.53	183.00	186.00	3	1	2
23	198.67	2.89	197.00	202.00	3	1	2
24	208.00	3.56	205.00	213.00	4	1	3
25	214.00		214.00	214.00	1	0	1
26	229.00	5.66	225.00	233.00	2	1	1
27	240.33	1.15	239.00	241.00	3	3	0
28	249.50	0.71	249.00	250.00	2	0	2
29	253.00	0.00	253.00	253.00	2	0	2
30	262.67	0.58	262.00	263.00	3	2	1
Total					37	16	21

**Fig. 1:**
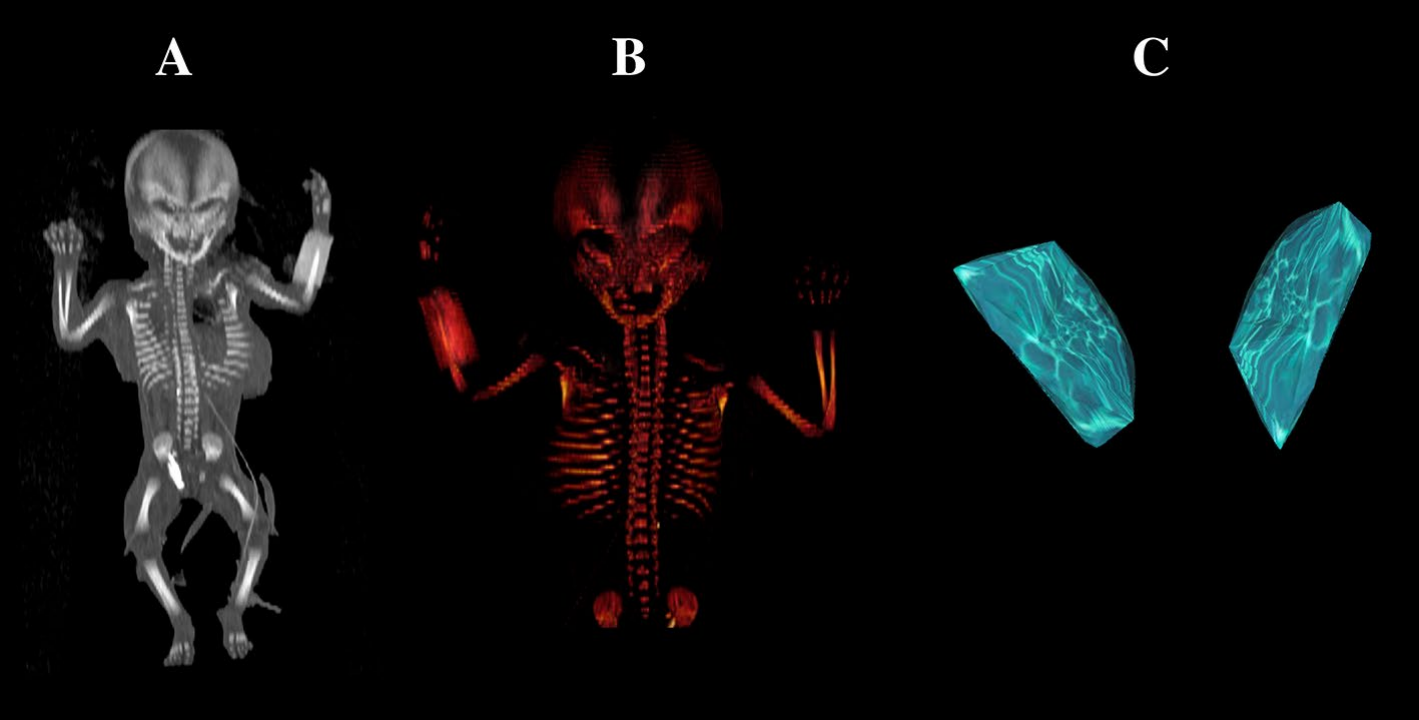
A female human fetus aged 21 weeks in the frontal projection (**a**), its skeletal reconstruction in frontal projection (**b**), 3D reconstruction of the right and left primary ossification center of the frontal squama (**c**) using Osirix 3.9 MD

**Fig. 2:**
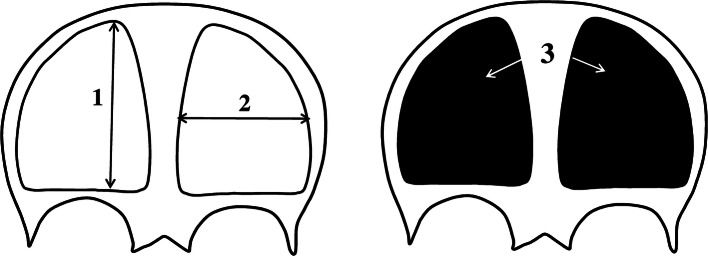
Measurement scheme of the primary ossification center of the frontal squama in the frontal plane: 1, vertical diameter; 2, transverse diameter; 3, projection surface area

Using the Siemens–Biograph 128 mCT scanner (Siemens Healthcare GmbH, Erlangen, Germany) located at Department of Positron Emission Tomography and Molecular Imaging (Oncology Center, Collegium Medicum of the Nicolaus Copernicus University, Bydgoszcz, Poland), scans of fetuses in DICOM formats were acquired at 0.4 mm intervals (Fig. 1). The gray scale of achieved CT pictures expressed in Hounsfield units (HU) varied from − 275 to − 134 for a minimum, and from + 1165 to + 1558 for a maximum. Therefore, the window width (WW) altered from 1.404 to 1.692, and the window level (WL) varied from + 463 to + 712. The specifics of the imaging protocol were presented by the following: mAs 60, kV 80, pitch 0.35, FoV 180, rot. time 0.5 s., while the specifics of CT data were: slice thickness 0.4 mm, image increment 0.6 mm, and kernel B45 f-medium. Measurements for each frontal bone were conducted in a specific order (Fig. 2). In each fetus, the assessment of the linear parameters, projection surface area and volume of the ossification center of the frontal squama was carried out. Despite the cartilaginous stage of development, morphometric analysis regarding its vertical and transverse diameters and volume was feasible, as the contours of the entire bone were already evidently visible [3, 6].

Measurements of the primary ossification center of the frontal squama (right and left) included:

1. vertical diameter, based on the determined distance between its proximal and distal borderlines in the frontal plane (Fig. 2);
2. transverse diameter, based on the determined distance between its medial and lateral borderlines in the frontal plane (Fig. 2);
3. projection surface area, based on the outlined area occupied by the ossification center of the frontal squama in the frontal plane (Fig. 2);
4. volume, calculated using advanced diagnostic imaging tools for 3D reconstruction, taking into account position and the absorption of radiation by bone (Fig. 1c).

The results of the investigation were statistically analyzed. Distribution of variables was checked using the Shapiro–Wilk (*W*) test. The homogeneity of variance was checked using Fisher’s test. The results were expressed as arithmetic means ± standard deviations (SD). To compare the means, Student’s *t* test for independent variables and one-way analysis of variance post hoc Tukey’s test were used. If no similarity of variance occurred, the non-parametric Kruskal–Wallis test was used. The characterization of developmental dynamics of the analyzed parameters was based on linear and curvilinear regression analysis. The match between the numerical data and computed regression curves was evaluated based on the coefficient of determination (*R*^2^). Correlations between the variables were also determined using Pearson’s linear correlation coefficient (*r*).

## Results

Mean values and standard deviations of the analyzed parameters of the left and right primary ossification center of the frontal squama in human fetuses at the analyzed gestational stages have been presented in Tables 2 and 3 for the vertical and transverse diameters, projection surface area and volume.

**Table 2:** Vertical and transverse diameters, projection surface area and volume of the right primary ossification center of the frontal squama in the human fetus

Gestational age (weeks)	Number of fetuses	Primary ossification center of the right frontal squama							
		Vertical diameter (mm)		Transverse diameter (mm)		Projection surface area (mm^2^)		Volume (mm^3^)	
		Mean	SD	Mean	SD	Mean	SD	Mean	SD
18	3	21.13	0.50	18.23	0.12	338.57	25.54	407.47	18.17
19	4	22.08	0.17	18.87	0.20	365.13	4.33	433.82	9.53
20	4	22.93	0.28	20.13	0.29	401.75	6.99	469.73	6.40
21	3	23.25	0.04	20.97	0.35	415.00	3.32	492.34	5.07
22	3	23.53	0.06	21.85	0.04	436.02	0.24	518.79	1.98
23	3	23.94	0.12	22.13	0.06	437.66	0.30	534.57	3.52
24	4	25.04	0.91	23.58	0.61	555.83	63.70	662.85	80.38
25	1	26.50	–	24.34	–	638.90	–	779.22	-
26	2	27.09	0.22	25.29	0.55	655.15	1.34	809.61	2.26
27	3	28.63	0.35	26.83	0.15	713.33	22.51	935.89	41.46
28	2	29.66	0.37	27.61	0.83	737.65	5.16	1032.69	11.54
29	2	31.93	0.95	28.33	0.04	771.10	0.14	1085.54	8.76
30	3	33.23	0.42	28.48	0.11	811.97	20.53	1136.59	45.02

**Table 3:** Vertical and transverse diameters, projection surface area and volume of the left primary ossification center of the frontal squama in the human fetus

Gestational age (weeks)	Number of fetuses	Primary ossification center of the left frontal squama							
		Vertical diameter (mm)		Transverse diameter (mm)		Projection surface		Volume (mm^3^)	
						area (mm^2^)			
		Mean	SD	Mean	SD	Mean	SD	Mean	SD
18	3	20.37	1.07	18.27	0.68	334.03	25.07	402.71	19.03
19	4	21.30	0.28	18.97	0.17	366.53	6.29	442.23	10.71
20	4	22.68	0.39	20.05	0.30	403.10	2.90	477.42	12.80
21	3	23.10	0.05	20.97	0.21	413.40	2.95	494.20	2.26
22	3	23.43	0.06	21.86	0.19	430.62	0.24	515.12	6.36
23	3	23.90	0.06	22.23	0.04	432.26	0.30	531.81	0.78
24	4	24.95	1.06	23.53	0.85	558.38	66.39	651.06	72.59
25	1	27.04	–	25.20	–	643.90	–	767.62	–
26	2	27.48	0.40	25.49	0.13	650.10	0.00	797.82	20.93
27	3	29.00	0.30	27.57	0.57	711.27	22.44	950.55	12.36
28	2	29.73	0.01	28.05	0.21	734.05	6.58	994.03	38.91
29	2	31.73	0.95	28.63	0.52	770.65	2.05	1059.72	41.00
30	3	33.13	0.25	30.43	0.95	812.67	20.81	1156.91	47.74

The statistical analysis revealed neither significant sex nor laterality differences, which allowed us to compute one growth curve for each analyzed parameter. The developmental dynamics of the vertical diameter followed a quadratic function, while that of the transverse diameter followed a linear function.

**Fig. 3:**
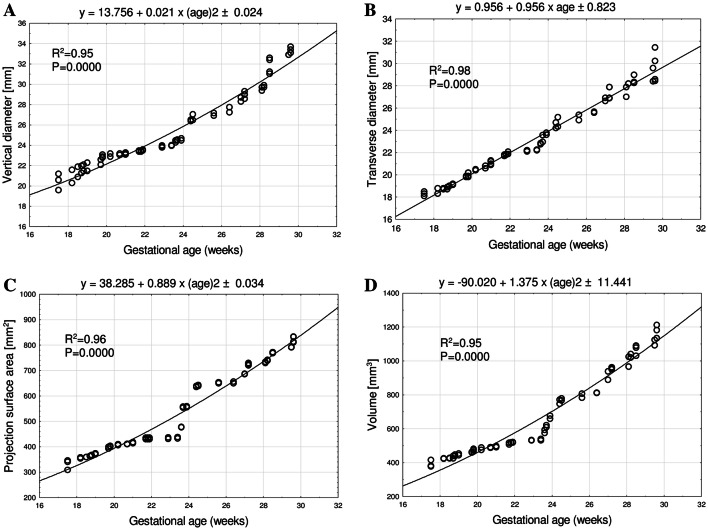
Regression lines for vertical diameter (**a**), transverse diameter (**b**), projection surface area (**c**), and volume (**d**) of the primary ossification center of the frontal squama

The mean vertical diameter of the primary ossification center of the frontal squama in the fetal age range of 18–30 weeks was between 21.30 ± 0.50 and 33.23 ± 0.42 mm on the right, and between 20.37 ± 1.07 and 33.13 ± 0.25 mm on the left, following the quadratic function: *y* = 13.756 + 0.021 × (age)^2^ ± 0.024 (*R*^2^ = 0.95) (Fig. 3a).

The mean transverse diameter of the primary ossification center of the frontal squama at the fetal ages of 18–30 weeks ranged from 18.23 ± 0.12 to 28.48 ± 0.11 mm on the right, and from 18.27 ± 0.68 to 30.43 ± 0.95 mm on the left, following the linear function: *y* = 0.956 + 0.956 × age ± 0.823 (*R*^2^ = 0.98) (Fig. 3b).

The mean projection surface area of the primary ossification center of the frontal squama ranged from 338.57 ± 25.54 mm^2^ at 18 weeks of gestation to 811.97 ± 20.53 mm^2^ at 30 weeks of gestation on the right, and from 334.03 ± 25.07 to 812.67 ± 20.81 mm^2^, respectively, on the left, following the quadratic function: *y* = 38.285 + 0.889 × (age)^2^ ± 0.034 (*R*^2^ = 0.96) (Fig. 3c).

The mean volume of the primary ossification center of the frontal squama in the fetal age range of 18–30 weeks was between 407.47 ± 18.17 and 1136.59 ± 45.02 mm^3^ on the right, and between 402.71 ± 19.03 and 1156.91 ± 47.74 mm^3^ on the left, following the quadratic function of age: *y* = 90.020 + 1.375 × (age)^2^ ± 11.441 (*R*^2^ = 0.95) (Fig. 3d).

## Discussion

The primary ossification of the frontal bone begins between weeks 8 and 11 of gestation from an ossification center located in the supraorbital region, in which the frontal eminences develop later [7–9, 11, 13, 18]. In the first trimester of pregnancy, ossification simultaneously progresses in the medial and lateral directions, which leads to the formation of the frontal (metopic) suture in the anterior midline of the frontal bone in the first year of postnatal life [17]. In turn, ossification in the second trimester occurs radially upwards, and the frontal suture becomes apparent throughout the surface of the frontal squama.

In this study, involving fetuses between 18 and 30 weeks of age, CT allowed an accurate visualization of the primary ossification center of the frontal squama in all examined fetuses.

From the third trimester of pregnancy, closure of the frontal suture begins from the glabella region towards the anterior fontanelle. Fusion of the two frontal squamae begins in the glabella region at approx. 16 weeks and ends at approx. 32 weeks of gestation [4, 7, 8, 17]. The frontal suture joins both frontal squamae and is physiologically the first osseous connection undergoing closure [19]. Vu et al. [22] showed that in a group of 159 infants aged 1 to 27 months, full closure of the frontal suture occurred in the 3rd month of life in 33% of infants, in the 5th month of life in 60% of infants, and by the 9th month of life in the remaining infants.

Secondary paired ossification centers appear in the region of the zygomatic process, nasal spine and trochlear fovea, and fuse by the end of the 7th month of life [11].

Morimoto et al. [16], using CT to examine formalin-fixed fetal material, studied changes in the shape of skull bones in human fetuses. They demonstrated a lack of sexual dimorphism in the prenatal period. Similarly, in our study, no sex differences in respect to the frontal bone were found.

Mandarim-de-Lacerda and Alves [14] weighed each bone of the skull. They noticed that the facial skeleton bones (vomer, palatine bone, mandible and maxilla) grew in a manner different to that of the neurocranial bones (sphenoid, ethmoid, frontal, occipital, parietal, temporal). The growth of the neurocranial bones was much faster than that of the facial skeleton bones. Moreover, the fastest growth in the neurocranium was noted for the bones forming the calvaria.

This paper is the first report about the morphometric analysis of the primary ossification center of the frontal squama in human fetuses with mathematical models describing its growth. The vertical diameter, projection surface area and volume of the primary ossification center of the frontal squama increased with fetal age following the quadratic functions: *y* = 13.756 + 0.021 × (age)^2^ ± 0.024 for the vertical diameter, *y* = 38.285 + 0.889 × (age)^2^ ± 0.034 for projection surface area, and *y* = 90.020 + 1.375 × (age)^2^ ± 11.441 for volume. The study also revealed that the transverse diameter of the primary ossification center of the frontal squama increased in a manner directly proportionate to fetal age, following the linear function: *y* = 0.956 + 0.956 × age ± 0.823.

Unfortunately, a lack of numerical data concerning the primary ossification center of the frontal squama in the medical literature limits a more detailed discussion on this topic.

The dimensions of the primary ossification center of the frontal squama obtained in the present study may be potentially useful in diagnosing skeletal dysplasias that are often characterized by a disrupted or completely halted growth of the frontal bone in the fetus. Since skeletodysplasias involve other bones of the cranial vault and their ossification points along with development of the neural tube, we are now planning to perform adequate quantitative studies of the parietal and occipital bones. One of congenital skull defects detected in a routine ultrasound examination is craniosynostosis, i.e. premature closure of one or several sutures, which leads to skull deformations. An isolated form of craniosynostosis is trigonocephaly caused by a premature closure of the frontal suture, which results in a delayed growth of the frontal bone and leads to skull deformation with characteristic bone hyperplasia in the midline, the narrowed anterior cranial fossa and increased biparietal diameter [17, 19]. Arrested growth of the anterior cranial fossa can cause increased intracranial pressure leading to impaired psychomotor function and neurological disorders [19]. The most distinguishing symptom of the premature closure of the frontal suture is an abnormal shape of the skull resulting from limited growth in the direction perpendicular to the closed suture and a compensative increase in growth in the parallel direction [19].

In a normally developing fetus, the distance between the two frontal squamae at the level of the cave of septum pellucidum decreases with fetal age from 2.2 mm in week 16 to 0.9 mm in week 32 of gestation [7]. Faro et al. [8] demonstrated that in fetuses with Apert syndrome, the distance between the two parts of the frontal bone measured at the level of the cave of septum pellucidum was 15–23 mm instead of the normal 1–2 mm. Apert syndrome is characterized by craniosynostosis and bilateral syndactyly of the hands and feet. The premature abnormal ossification of both lambdoid sutures typical of Apert syndrome results in a reduced antero-posterior dimension of the skull with a delay in the closure of the anterior and posterior fontanelles that may gradually increase in the first months after birth. The frontal region remains flattened, with shallow orbit and hypertelorism, and the palpebral fissures directed diagonally downwards [10].

In turn, non-closure of the frontal squamae leads to metopism. Such skulls are characterized by a wide forehead, increased distance between the orbits and greater frontal curvature [17].

In holoprosencephaly, which is associated with microcephaly, premature ossification of the frontal ossification centers and closure of the frontal suture occur, probably due to the lack of cephalic growth and the absence of the stimulus that stretches the suture [7]. In many fetuses with abnormal development of the frontal suture, developmental disorders of the encephalon can be observed. Chaoui et al. [4] demonstrated that in fetuses with holoprosencephaly and abnormal development of the corpus callosum, premature closure of the frontal suture occurs, while in fetuses with cerebellar abnormalities, ossification of the frontal bone is delayed.

Routine pre-natal examinations include ultrasound which is insufficient when skull abnormalities are suspected. To make a correct diagnosis of a skeletal dysplasia, the examination needs to involve more accurate diagnostic imaging methods, such as CT or MRI [17, 21, 22].

Van Zalen-Sprock et al. [20] demonstrated that the ossification centers in the human fetus can be first observed using X-ray due to the radiation absorption capacity of bone. Radiographic examinations of the skull are most commonly used in the case of suspicion of skeletal dysplasia, but also for the assessment of head shape or perinatal injury. When using transvaginal ultrasound, a method safer for the human fetus, ossification centers are visible in the same period or approximately 1 week later.

Victoria et al. [21] and Cassart et al. [2] demonstrated that the use of 3D CT to detect skeletal dysplasias provides a higher imaging precision than 2D ultrasound. Unfortunately, the sensitivity of ultrasound when diagnosing skeletal dysplasia is only 40–60%, so low-dose CT may play a propitious role in cases of suspected fetal skeletal dysplasia [19]. Victoria et al. [19] compared both the effectiveness and usefulness of US and CT in the prenatal diagnostics of skeletodysplasias and demonstrated that among the 21 cases included in the study, in only 5 cases CT and ultrasonic findings were analogue, while in 17 cases CT unveiled novel osseous findings, not detected by ultrasound. Among 218 measurements carried out, a total of 4 erroneous findings referred to CT, and as many as 19 erroneous findings referred to ultrasonography. Computed tomography, due to its high sensitivity and reproducibility, allows more detailed and unambiguous diagnostics, as well as 3D imaging and intracranial assessment. The advantage of fetal CT examinations results from the fact that it can be entirely reinterpreted at any given time with no loss of imaging details after the study is finished. Besides, CT eliminates the overlap of anatomical structures and allows for easy distinction between different body tissues [12, 15]. To our opinion, it should be emphasized that CT examination cannot be used to the evaluation of minor osseous abnormalities. Contrariwise, it may be performed as a complementary method to ultrasonography in the diagnosis of severe and potentially lethal abnormalities. As reported by Macé et al. [12], in the diagnosis of fetal skeletodysplasias a helical CT examination may be useful from week 26 of gestation and should be performed in cases with severe micromelia below the 3rd percentile and for those ≤ 10th percentile associated with another bone sign. According to these authors, the fetal age above 26 weeks is a period of pregnancy which ensures additional safety because of the development of potential exposed organs. In the third trimester of pregnancy, the ossification process is satisfactory to correctly analyze CT images. Simultaneously, it is more difficult to obtain adequate viewing planes in 3-dimensional ultrasonography. A factor that limits the applicability of CT examinations is the lack of numerical data describing the fetal skeletal system at the defined weeks of pregnancy in comparison with routine ultrasound examinations. Currently, magnetic resonance imaging (MRI) has become a clinical complement for ultrasound and is the most accurate and safest diagnostic tool used to assess fetal anatomy in both prenatal and post-mortem examinations. MRI is extremely indispensable in the 2nd and 3rd trimesters when ultrasound imaging is either equivocal or limited by, e.g., low amniotic fluid volume (oligohydramnios) or inadequate fetal positioning in respect to the plane of contact of the transducer [5]. Currently, MRI is used in examinations when congenital defects of the skeletal and central nervous systems, as well as thoracic and abdominal organs are suspected [1].

The main limitation of this study was a relatively narrow fetal age group, ranging from the 18th to the 30th week of pregnancy, and a small number of cases, including 37 human fetuses.

## Conclusions

1. The morphometric characteristics of the primary ossification center of the frontal squama display neither sex nor laterality differences.
2. The ossification center of the frontal squama grows following a quadratic function in its vertical diameter, projection surface area and volume, and linearly in its transverse diameter.
3. Our findings for the primary ossification center of the frontal squama may be conducive in monitoring normal fetal growth and screening for inherited faults and anomalies of the skull in human fetuses.
4. More novel studies about the frontal squama growth should be undertaken to help gain more insight into understanding of occurrence of skeletodysplasias.
